# Background Rates of Adverse Pregnancy Outcomes for Assessing the Safety of Maternal Vaccine Trials in Sub-Saharan Africa

**DOI:** 10.1371/journal.pone.0046638

**Published:** 2012-10-04

**Authors:** Lauren A. V. Orenstein, Evan W. Orenstein, Ibrahima Teguete, Mamoudou Kodio, Milagritos Tapia, Samba O. Sow, Myron M. Levine

**Affiliations:** 1 Emory University School of Medicine, Atlanta, Georgia, United States of America; 2 Centre pour le Développement des Vaccins-Mali, Bamako, Mali; 3 Gabriel Touré Teaching Hospital, Department of Obstetrics and Gynecology, Bamako, Mali; 4 Center for Vaccine Development, University of Maryland School of Medicine, Baltimore, Maryland, United States of America; Aeras, United States of America

## Abstract

**Background:**

Maternal immunization has gained traction as a strategy to diminish maternal and young infant mortality attributable to infectious diseases. Background rates of adverse pregnancy outcomes are crucial to interpret results of clinical trials in Sub-Saharan Africa.

**Methods:**

We developed a mathematical model that calculates a clinical trial's expected number of neonatal and maternal deaths at an interim safety assessment based on the person-time observed during different risk windows. This model was compared to crude multiplication of the maternal mortality ratio and neonatal mortality rate by the number of live births. Systematic reviews of severe acute maternal morbidity (SAMM), low birth weight (LBW), prematurity, and major congenital malformations (MCM) in Sub-Saharan African countries were also performed.

**Findings:**

Accounting for the person-time observed during different risk periods yields lower, more conservative estimates of expected maternal and neonatal deaths, particularly at an interim safety evaluation soon after a large number of deliveries. Median incidence of SAMM in 16 reports was 40.7 (IQR: 10.6–73.3) per 1,000 total births, and the most common causes were hemorrhage (34%), dystocia (22%), and severe hypertensive disorders of pregnancy (22%). Proportions of liveborn infants who were LBW (median 13.3%, IQR: 9.9–16.4) or premature (median 15.4%, IQR: 10.6–19.1) were similar across geographic region, study design, and institutional setting. The median incidence of MCM per 1,000 live births was 14.4 (IQR: 5.5–17.6), with the musculoskeletal system comprising 30%.

**Interpretation:**

Some clinical trials assessing whether maternal immunization can improve pregnancy and young infant outcomes in the developing world have made ethics-based decisions not to use a pure placebo control. Consequently, reliable background rates of adverse pregnancy outcomes are necessary to distinguish between vaccine benefits and safety concerns. Local studies that quantify population-based background rates of adverse pregnancy outcomes will improve safety assessment of interventions during pregnancy.

## Introduction

Maternal and neonatal mortality in Sub-Saharan Africa present a major barrier to achievement of Millennium Development Goals 4 and 5. Sub-Saharan Africa accounts for 52-57% of all maternal deaths worldwide and contains 23 of the 27 countries with neonatal morality rates greater than 30 per 1000 live births [1], [2], [3]. Maternal immunization has gained traction as one strategy to diminish maternal and infant mortality attributable to infectious diseases [4], [5], [6], [7], [8], and may also reduce prematurity and low birth weight [9], [10]. Vaccines protect pregnant women who are more susceptible to severe disease [4], [11], mitigate harms of infection on the developing fetus [8], [9], [12], [13], and passively immunize infants too young to be successfully vaccinated [14], [15]. Maternal immunization may protect young infants directly through placental transport of IgG antibodies (with higher antibody titers found in infants of increasing gestational age) and indirectly through herd immunity [16], [17], [18].

Vaccination to prevent tetanus, influenza, hepatitis B, and invasive meningococcal disease is currently recommended for pregnant women in high-income countries [19]. Maternal immunization with tetanus toxoid to prevent both maternal and neonatal tetanus is already widespread in developing countries, and concerted efforts are exploring the feasibility of administering additional vaccines (e.g., influenza, meningococcal, yellow fever). Before maternal immunization can become a mainstream public health intervention, convincing data must document each additional vaccine's safety for the pregnant woman and her developing fetus and newborn. Several randomized controlled trials of maternal immunization with influenza vaccine are underway in developing countries, including in Sub-Saharan Africa. To provide a benefit to all study participants, such trials often administer to controls a licensed vaccine unrelated to the outcome of interest rather than a placebo. In this situation, it may be difficult to distinguish whether a difference in outcomes between trial arms represents benefit from the interventions with superior results or harms of interventions with poorer outcomes. For example, if a two-arm trial demonstrates a significant decrease in the rate of small for gestational age (SGA) in vaccine group A compared to group B, does this indicate that vaccine A has reduced the incidence of SGA or that vaccine B has increased the incidence of SGA [15]? Consequently, knowledge of the background rates of adverse pregnancy outcomes is crucial to interpret the results of clinical trials in Sub-Saharan Africa. Further adaptations of these data to yield the number of expected adverse events based on the amount of person-time observed before and after delivery can provide an important context to inform interim decisions made by independent Data Safety Monitoring Boards (DSMB) and researchers involved with clinical trials in such vulnerable populations.

This review seeks to assemble and synthesize available data on pregnancy outcomes in Sub-Saharan Africa to provide a context for assessing the safety of maternal immunization at interim stages during a clinical trial as well as after follow-up is complete. We present expected rates of maternal mortality, severe acute maternal morbidity (SAMM), neonatal mortality, low birth weight (LBW), and major congenital malformations (MCM) in a hypothetical cohort of pregnant women enrolled in a clinical trial in Sub-Saharan Africa.

## Methods

### Maternal Mortality, Stillbirths, and Neonatal Mortality

We used a mathematical model to develop interim estimates of maternal and neonatal deaths over the course of a clinical trial. Maternal mortality was defined as death from direct or indirect obstetric causes during pregnancy or <42 days after pregnancy termination [2]. Stillbirths, early neonatal deaths, and late neonatal deaths were defined as death at birth, death of a liveborn infant within the 1^st^ week of life, and death of a liveborn infant between 1 and 4 weeks of life, respectively [20].

In an interim analysis of vaccine safety by an independent DSMB during a clinical trial of maternal immunization, the expected number of maternal deaths depends on the person-time observed during different risk windows. We considered three periods: pregnancy, within 24 hours of delivery, and the first 42 days post-partum. Large prospective cohort studies in Sub-Saharan Africa were combined by random-effects meta-analysis to determine the proportions of maternal deaths occurring during each interval [21], [22], [23], [24], [25], [26], [27]. The expected number of maternal deaths was thereby calculated using an observation-time model:
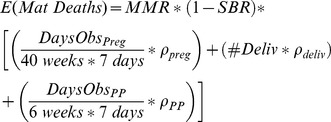
(1)


Where *E(Mat Deaths)* is the expected number of maternal deaths at a particular stage of recruitment; *MMR* is the maternal mortality ratio; *SBR* is the stillbirth rate; *DaysObs_Preg_*, *#Deliv*, and *DaysObs_PP_* are the total number of days of observation during pregnancy, the number of deliveries, and the days of observation during the post-partum period; *ρ_preg_*, *ρ_deliv_*, and *ρ_PP_* represent the proportion of maternal deaths occurring before delivery, within 24 hours of delivery, and in the post-partum period, respectively.

This equation makes the following three assumptions, all of which bias towards underestimating the expected background number of deaths and hence create more stringent thresholds for evaluating vaccine safety.

1. The risk of maternal death before delivery is evenly distributed throughout pregnancy.
2. The risk of maternal death between 24 hours after delivery and 42 days post-partum is also uniformly distributed.
3. The maternal mortality ratio multiplied by 1 minus the stillbirth rate approximates the ratio of maternal deaths per 100,000 total births. We also assume that each birth contributes 46 weeks of maternal person-time, yielding an incidence of maternal deaths per 100,000 * 46 maternal weeks. The calculated incidence is lower than the true incidence of maternal deaths because women who die before finishing 6 weeks post-partum contribute fewer than 46 maternal weeks of person-time.

Similarly, the expected number of stillbirths and neonatal deaths at an interim analysis would depend on the number of deliveries and the amount of person-time observed:
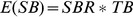
(2)

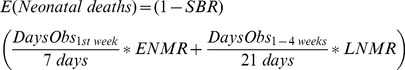
(3)


Where *E(SB)* and *E(Neonatal Deaths)* are the expected number of stillbirths and neonatal deaths; *TB* is the sum of live-births and stillbirths that have occurred; *DaysObs_1st week_* and *DaysObs_1–4 weeks_* are the number of infant-days of observation during the first week of life and between 1 and 4 weeks of life, respectively.

Equation (3) assumes a uniform risk of death throughout the 1^st^ week of life and a uniform risk of death between 1 and 4 weeks of life. We did not separate neonatal deaths in the 1^st^ day of life from other early neonatal deaths as reliable country-specific early neonatal mortality rates have been described [1], but reliable proportions of deaths in the 1^st^ day of life by country are not available.

We compared the observation-time model estimates of maternal and neonatal deaths to a crude calculation based on the number of live births:
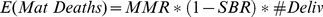
(4)

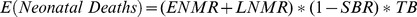
(5)


Statistical calculations were performed in R Version 2.12.1, WinBUGS Version 1.4, and StatsDirect Version 2.7.8.

### Guidelines for Systematic Reviews

Systematic reviews were conducted on the following pregnancy outcomes in Sub-Saharan Africa: severe acute maternal morbidity (SAMM), low birth weight (LBW), small for gestational age (SGA), prematurity, and major congenital malformations (MCM). For each systematic review, we searched the Medline electronic database for English, French, and Portuguese language publications in peer-reviewed journals. Prospective cohorts, retrospective cohorts, and cross-sectional studies with defined catchment populations were included. Studies in which all data were collected before 1991 were excluded except in the review of congenital malformations. Studies with <100 live births in Sub-Saharan Africa were excluded. Regions of Sub-Saharan Africa were defined by the Global Burden of Disease Study [28]. The reference lists of identified articles were reviewed to locate further eligible studies. All database searches were updated in March 2012.

We followed the Preferred Reporting Items for Systematic Reviews and Meta-Analyses guidelines [29]. All data were extracted independently by two authors, with disagreements mitigated by a third author. No research ethics board approval was obtained as data were extracted from published reports. No separate review protocol was developed beyond what is specified here. Bias was not assessed for individual studies once included.

### Severe Acute Maternal Morbidity

Literature search terms were “severe maternal morbidity OR near miss” along with the name of each country in Sub-Saharan Africa. The principal outcome measure was the incidence of severe acute maternal morbidity (SAMM) or near-miss cases, defined as direct or indirect obstetric complications that threaten the woman's survival but do not lead to her death [30]. Studies were included if they contained a definition distinguishing severe maternal morbidity from all maternal morbidity [31] and provided a catchment population. Studies were excluded if they did not provide sufficient data to calculate the incidence of SAMM or if it was not possible to separate near-miss events from maternal deaths, as the latter are accounted for in the maternal mortality section. Where possible, the number of deliveries was used as the denominator. We considered 6 categories of SAMM: hemorrhage, hypertensive disease of pregnancy, dystocia, infections, anemia, and other.

### Low birth weight and prematurity

Literature search terms were “low birth weight OR prematurity OR small for gestational age,” along with the name of each country in Sub-Saharan Africa. LBW was defined as birth weight <2.5 kg measured by the study team within 7 days of life or extracted from official birth records. Prematurity was defined as estimated gestational age at delivery <37 weeks as determined by ultrasound, last menstrual period, or validated exam within 7 days of life [32], [33], [34], [35]. Where possible, outcomes were extracted for singleton live-births.

### Congenital Malformations

Literature search terms were “major congenital abnormalities OR congenital malformations [title] OR congenital anomalies [title]” along with the name of each country in Sub-Saharan Africa. The principal outcome measure was the incidence of MCM in liveborn infants, defined as structural defects of the body and/or the organs that affect viability or quality of life and require medical intervention [36]. Studies were excluded if the incidence of MCM could not be calculated or if malformations in stillborn infants could not be separated. Malformations were classified by organ system using International Classification of Diseases (ICD)-10 codes [37].

## Results

### Maternal and Neonatal Mortality

**Figure 1:**
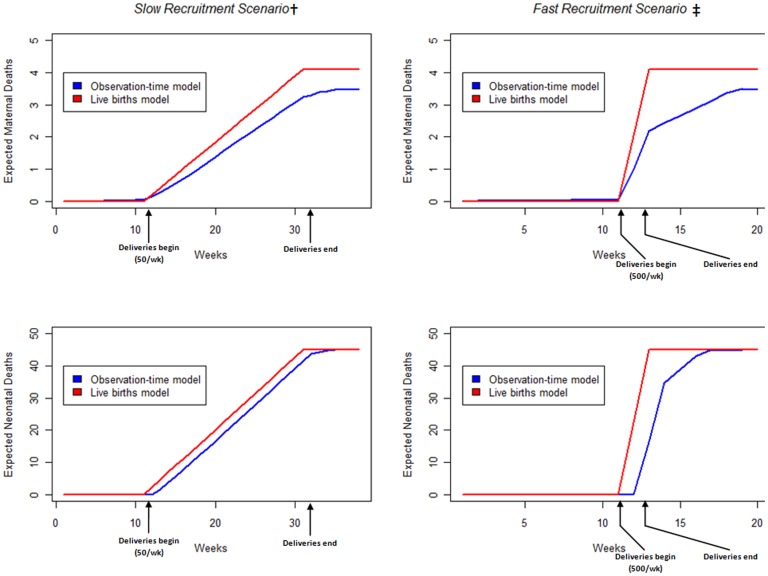
Observation-time model vs. Live births model to calculate expected background maternal and neonatal deaths over the course of a clinical trial in 1000 pregnant women in Mali. Assuming a maternal mortality ratio of 418.8 (327.5–519.8) per 100,000 live births, stillbirth rate of 23 (18–42) per 1,000 total births, early neonatal mortality rate of 33.5 (28.1–39.0) per 1,000 live births, and late neonatal mortality rate of 12.4 (9.9–15.5) per 1,000 live births estimated for Mali by recently published systematic analyses [3], [44], [45]. We assume women are recruited at 28 weeks gestation and deliver exactly 12 weeks later. † 50 pregnant women recruited each week over 20 weeks. ‡500 pregnant women recruited each week over 2 weeks.

We compared the expected background rates of maternal and neonatal deaths over the course of a hypothetical clinical trial of 1000 pregnant women in Mali given by the observation-time model to a crude calculation based on live births (Figure 1). In the observation-time model, the proportions of maternal deaths occurring before, during, and after delivery were modeled as 18.6% (CI: 14–24), 44.9% (CI: 35–53), and 36.5% (CI: 27–47), respectively [21], [22], [23], [24], [25], [26], [27] (Table S1). Anticipated maternal and neonatal deaths were consistently lower in the observation-time model than the corresponding live births calculation. This effect was magnified immediately after a large number of deliveries. At the end of the trial, expected maternal deaths were also lower in the observation-time model because women were not observed for the entirety of pregnancy, and thus had already experienced part of the risk window for maternal death by the time of study inclusion.

### Severe Acute Maternal Morbidity

**Figure 2:**
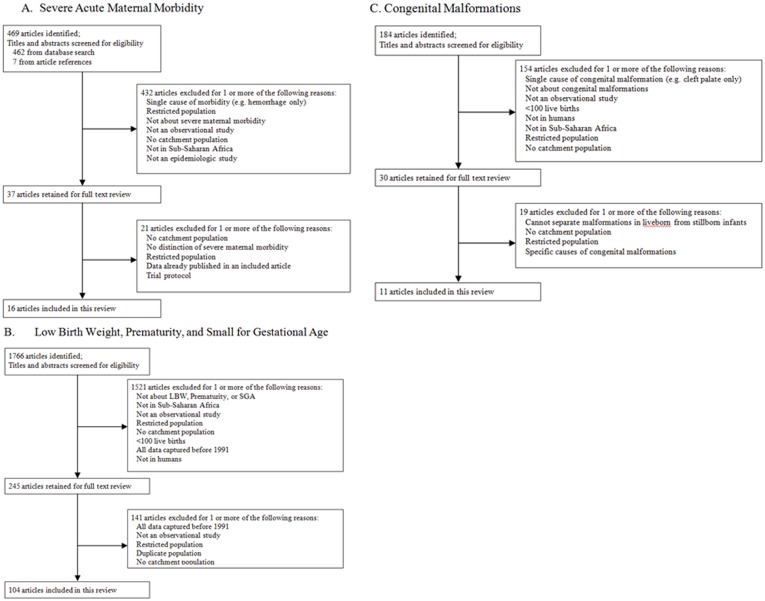
Summary of literature search and study selection. A. Severe Acute Maternal Morbidity. B. Low Birth Weight, Prematurity, and Small for Gestational Age. C. Congenital Malformations.

We screened 469 titles and abstracts and 37 full texts and included 16 studies of SAMM in Sub-Saharan Africa (Figure 2A). The median incidence of SAMM per 1,000 total births was 40.7 (IQR: 10.6–73.3) (Table 1). SAMM definitions were notably heterogeneous but were categorized as either organ failure/management-based [38] or disease-specific [39]. Among the 5 studies with organ failure/management-based definitions, the median incidence of SAMM was significantly lower than the 11 studies with disease-specific definitions (6.9 vs. 56.8 per 1,000 total births, p = .002), which may reflect more stringent requirements in the organ failure/management approach. Mortality indices of studies with organ failure/management-based criteria were substantially though not significantly higher (median 16.5% vs. 9.1%, p = .052). Studies conducted in West Africa had significantly higher SAMM incidences than those outside of West Africa (median 92.6 vs. 11.3, p = 0.001), even when restricting to studies with a disease-specific definition (median 92.6 vs. 31.5, p = 0.02). The most common causes of severe maternal morbidity were hemorrhage, dystocia, and hypertensive disorders, accounting for 34%, 22%, and 22% of all events, respectively.

**Table 1:** The incidence of severe acute maternal morbidity in 16 studies in Sub-Saharan Africa published between 1995 and 2012.

Study Author	Country	Years	SAMM Definition	MI (%)	Site of case detection	Denominator	Size	#SAMM per 1000 Births	SAMM by Cause (%)					
									Hem	Dyst	HTN	Anem	Infect	Other
Okong [46]	Uganda	1999–2000	Organ failure/management	54	Urban and rural hospitals	Hospital births	55803 LB	**4.1**	42	21	7.9	0	18	11
Gandhi [47]	South Africa	—	Organ failure/management	—	Rural hospitals	Hospital and ancillary clinic births	5728 TB	**5.4**	19	6.5	52	0	9.7	13
Cochet [48]	South Africa	2000–2001	Organ failure/management	14	Urban hospitals	Hospital births	29832 TB	**6.9**	40	0	15	0	12	34
Van den Akker [49]	Malawi	2007–2009	Disease-Specific	12	Rural hospital	All facility deliveries in district	33254 D	**10.2**	32	11	20	0	32	5.1
Mantel [38]	South Africa	1996–1997	Organ failure/management	17	Urban hospitals	All deliveries in region	13429 D	**10.9**	26	0	26	0	20	29
Vandecruys [50]	South Africa	1997–1999†	Organ failure/management	16	Urban hospitals	Hospital births	26152 TB	**11.7**	18	0	40	0	13	28
Ali [51]	Sudan	2008–2010	Disease-Specific	16	Urban hospital	Hospital births	9578 TB	**21.4**	41	8	18	12	22	0
Mayi-Tsonga [52]	Gabon	2006	Disease-Specific	—	Urban hospital	Hospital births	4350 TB	**31.5**	82	0	14	0	4.4	0
Nyamtema [53]	Tanzania	2008–2010	Disease-Specific	9.9	Rural hospital	Hospital births	6572 TB	**49.8**	30	22	28	8.0	4.0	8.6
Prual [54]	6 countries (West Africa)	1994–1996	Disease-Specific	3.7	Predominantly urban communities	Pregnant women followed prospectively	20326 D	**52.4** *	50	34	10	0	1.0	4.0
Prual [55]	Niger	—	Disease-Specific	8.3	Urban hospitals	Hospital and ancillary clinic births	4081 TB	**56.8**	13	56	18	0	3.4	8.6
Gessessew [56]	Ethiopia	1993–2003	Disease-Specific	6.9	Urban hospital	Hospital births	7150 D	**60.0**	21	70	9.3	0	0	0
Lori [57]	Liberia	2008	Disease-Specific	19	Rural hospital	Hospital births	1386 TB	**86.6**	42	5	11	21	14	4.0
Filippi [39]	Benin, Côte d'Ivoire	1999–2001	Disease-Specific	6.5	Predominantly urban hospitals	Hospital births	27620 TB	**98.6**	34	15	26	19	5.4	0
Oladapo [58]	Nigeria	1999–2004	Disease-Specific	16	Urban hospital	Hospital deliveries	2577 D	**149.8**	31	20	30	9.0	11	0
Cham [59]	Gambia	2006	Disease-Specific	3.6	Urban and rural hospitals	Hospital births	3280 TB	**242.7**	20	26	26	17	1.4	10
						**Weighted Mean**		**46.4**	34	22	22	10.5	7.0	5.0

SAMM: Severe Acute Maternal Morbidity; MI: Mortality Index (# of maternal deaths divided by the sum of near-miss cases and maternal deaths) TB: Total births; D: Deliveries; Hem: Hemorrhage; Dyst: Dystocia (includes uterine rupture); HTN: Hypertensive diseases of pregnancy (severe pre-eclampsia and eclampsia); Anem: Anemia; Infect: Infection;

† Data from the year 2000 published by Vandecruys et al [50] were also published in Cochet et al [48]. In this table, those data were removed from Vandecruys et al [50] to avoid duplication.

* In Prual et al [54], 109 Cesarean sections performed for scarred uterus, fetal distress, and premature rupture of membranes that did not meet criteria for severe dystocia were subtracted from the total number of severe maternal morbidities as these ostensibly did not directly threaten the life of the mother.

### Low Birth Weight and Prematurity

**Figure 3:**
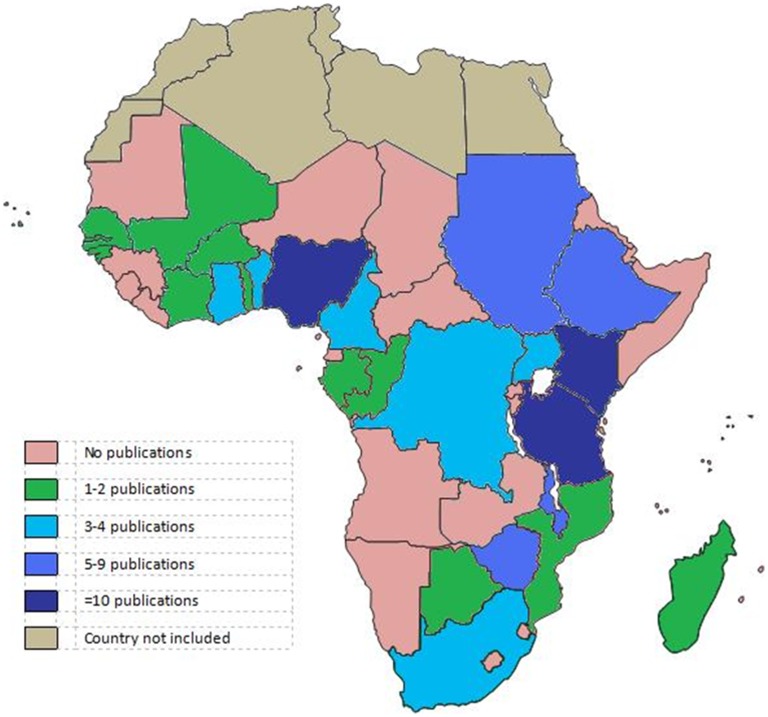
Publications by country on low birth weight, prematurity, and small for gestational age in sub-Saharan Africa.

We screened 1766 titles and abstracts and 245 full texts and included 104 studies in our review of LBW, prematurity, and SGA (Figure 2B). Studies originated from 26 sub-Saharan African countries; Nigeria, Kenya, and Ethiopia each contributed ≥10 studies while 4 other countries contributed 5–9 studies (Figure 3). The median incidence of LBW in 97 studies was 13.3% (IQR: 9.9–16.4), the median incidence of prematurity in 30 studies was 15.4% (IQR: 10.6–19.1), and the median incidence of SGA in 14 studies was 10.5% (IQR: 6.5–18.8) (Table S2). There were 53 perinatal surveys, 30 record reviews, and 21 cohort studies. Fifty-eight studies were performed in urban or semi-urban hospitals, 19 in clinics, 15 in rural hospitals, and the remaining 12 were population-based. Estimates of the incidence of LBW and prematurity and the mean birth weight did not vary significantly by study design, setting, region of sub-Saharan Africa, or method of gestational age assessment. The median proportion of SGA in 4 studies that defined SGA as <10^th^ percentile by standardized growth curve was significantly higher than 6 studies in which term-LBW was the only indicator of SGA (17.2% vs. 7.5%, p = 0.019).

### Major Congenital Malformations

We screened 184 titles and abstracts and 30 full texts and included 11 studies in our review of MCM among liveborn infants in Sub-Saharan Africa (Figure 2C). The median incidence per 1,000 live births was 14.4 (IQR: 5.5–17.6) (Table 2). Seven of the 11 studies were limited to physical examination findings at birth and did not have imaging support. The most common major malformations were found in the musculoskeletal (30%), central nervous (16%), and gastrointestinal systems (13%).

**Table 2:** The incidence of congenital malformations among liveborn infants in 11 studies in Sub-Saharan Africa published between 1966 and 2009.

Author	Country	Years	Study Design	Method of CM Detection	# LB	# Expected MCM per 1000 LB	Distribution of Major Congenital Malformations (%):									
							MSK	CNS	GI	GU	Chrom	CV	HEENT	Resp	Multip	Other
Ahuka [60]	Dem Rep Congo	1993–2001	Retrospective cohort, hospital based	Midwife exam at birth	8824	**4.1**	31	47	17	2.8	0	0	0	0	0	2.8
Sukhani [61]	Zambia	1976	Prospective cohort, hospital based	Physical exam at birth, imaging as indicated	17030	**5.3**	13	19	16	9.7	13	9.7	0	4.3	8.6	6.5
Embree [62]	Kenya	—	Prospective cohort, hospital based	Standardized exam at birth and 6 month intervals	183	**5.5**	—	—	—	—	—	—	—	—	—	—
Shija [63]	Zimbabwe	1984	Prospective cohort, hospital based	Physical exam at birth or pediatric surgery clinic	18033*	**7.0**	21	4.5	29	12	0	0	0	0	15	20
Delport [64]	South Africa	1986–1989	Prospective cohort, hospital based	Physician exam at birth	17351	**11.9**	18	19	9.2	7.8	14	15	1.0	0.5	3.9	11
Abudu [65]	Nigeria	1982–1983	Prospective cohort, hospital based	Physician exam at birth, autopsy	2912	**14.4**	—	—	—	—	—	—	—	—	—	—
Venter [66]	South Africa	1989–1992	Prospective cohort, hospital based	Standardized exam by geneticist at birth, lab tests and imaging as indicated	7617	**15.2**	23	28	5.2	8.6	18	0	0.9	0	7.8	7.8
Stevenson [67]	South Africa	1961–1964	Prospective cohort, hospital based	Standardized exam at birth	23675	**15.6**	—	—	—	—	—	—	—	—	—	—
Khan [68]	Zambia	1974–1975	Prospective cohort, hospital based	Physical exam at birth	8508	**17.6**	63	4.0	6.0	5.3	2.7	4.7	1.3	0	6.0	6.7
Gupta [69]	Nigeria	1964	Prospective cohort, hospital based	Standardized exam at birth	4054	**26.9**	39	14	19	5.5	0	10	7.3	0	0	5.5
Bakare [70]	Nigeria	2003–2004	Prospective cohort, outpatient delivery wards	Physical exam at birth	624	**36.9**	43	13	0	39	0	0	0	0	0	4.3
				**Weighted Mean**		**11.7**	**30**	**16**	**13**	**8.6**	**7.7**	**6.7**	**1.5**	**0.6**	**8.6**	**9.4**

CNS = Central Nervous System (ICD-10: Q00–Q07); Resp = Respiratory (ICD-10: Q30–Q34); CV = Cardiovascular (ICD-10: Q20–Q28); MSK = Musculoskeletal system (ICD-10: Q65–79); GI = Digestive system (ICD-10: Q35–Q45); GU = genital organs and urinary system (ICD-10: Q50–Q56, Q60–Q64); HEENT = Eye, ear, face, and neck (ICD-10: Q10–Q18); Chrom = Chromosomal abnormalities (ICD-10: Q90–Q99); Multip = Major congenital malformations in multiple systems.

* Denominator given in total births.

### Summary of Background Rates of Adverse Pregnancy Outcomes

Pregnancy outcomes including maternal deaths, SAMM, stillbirths, neonatal deaths, LBW, prematurity, and MCM are summarized for the 4 sub-regions of Sub-Saharan Africa (Table 3), and estimates of maternal deaths, stillbirths, neonatal deaths, LBW, and prematurity are presented for each country in sub-Saharan Africa (Table 4). In all categories except LBW and prematurity, anticipated adverse events were more frequent in West Africa than in the other 3 sub-regions. Variation was widest for severe maternal morbidity and major congenital malformations, the two categories for which the fewest data are available.

**Table 3:** Background rates of pregnancy outcomes by region of Sub-Saharan Africa.

*Region of Sub-Saharan Africa*	*Maternal deaths per 1000 Total Births* ¥	*SAMM per 1000 Total Births* †	*Stillbirths per 1000 Total Births*	*Early NND per 1000 Total Births* ¥	*Late NND per 1000 Total Births* ¥	*% LBW*	*% <37 wks*	*% Liveborn infants with MCM* †
Central	4.5 (3.7–5.3)	31.5 (—)	24.6 (12.3–52.4)	25.4 (22.6–28.3)	8.8 (3.1–5.0)	12.4 (10.3–34.2)	17.9 (2.3–21.3)	0.41 (—)
East	4.0 (3.7–4.4)	21.4 (10.2–59.4)	24.8 (16.3–43.9)	20.3 (19.1–21.5)	6.9 (6.3–7.6)	12.4 (6.3–37.1)	11.6 (3.4–20.3)	0.55 (0.53–1.76)
Southern	1.7 (1.4–2.0)	6.8 (5.2–11.0)	20.1 (13.4–32.7)	13.1 (12.2–14.3)	4.2 (3.7–4.9)	14.1 (6.0–20.3)	18.7 (17.3–20.1)	1.36 (0.70–1.56)
West	4.6 (4.2–5.1)	92.6 (56.8–242.7)	33.3 (20.3–58.8)	26.2 (24.1–28.4)	9.7 (8.7–10.7)	13.3 (5.5–29.0)	13.4 (5.3–30.5)	2.69 (1.44–3.69)

SAMM = Severe Acute Maternal Morbidity; NND = Neonatal Deaths; LBW = Low Birth Weight; MCM = Major Congenital Malformations.

† For Severe Maternal Morbidity, Low Birth Weight, Prematurity, and Major Congenital Malformations, the median and range of a systematic review is presented for the entire region. In Central Africa, only 1 data point was available for Severe Maternal Morbidity [52] and for Major Congenital Malformations [60], so no range was presented.

¥ Maternal and neonatal deaths were expressed as a fraction of total births by multiplying the maternal mortality ratio (maternal deaths/live births) and the early and late neonatal mortality ratios (neonatal deaths/live births) by 1 minus the stillbirth rate [44].

**Table 4:** Expected number of maternal and neonatal deaths and stillbirths for a hypothetical cohort of pregnant women corresponding to 1,000 births in Sub-Saharan Africa and the proportion of live-born infants expected to be low birth weight or premature.

	Maternal deaths per 1000 Total Births [1] ¥	Stillbirths per 1000 Total Births [44]	Early neonatal deaths per 1000 Total Births [1] ¥	Late neonatal deaths per 1000 Total Births [1] ¥	% LBW†	% <37 wks†
*Central Africa*						
Angola	3.3(2.1–4.5)	25.1(12–54)	28.0(24–33)	10.2(8.0–13)	**—**	**—**
Central African Republic	6.7(5.0–8.6)	24.2(12–49)	31.5(27–37)	12.9(11–16)	**—**	**—**
Congo (Brazzaville)					12.4 (—)	16.7 (—)
Congo (Dem Republic)	4.7(3.6–5.9)	25.6(14–55)	24.4(21–28)	8.2(6.7–9.7)	20.0 (10.5–34.2)	2.3 (**—**)
Equatorial Guinea	2.1(1.3–3.2)	16.9 (8–36)	35.1(29–42)	15.1(12–19)	**—**	**—**
Gabon	4.2 (3.1–5.4)	17.3 (9–38)	22.4 (19–26)	3.9 (3.1–5)	10.5 (10.3–10.7)	20.2 (19.1–21.3)
*East Africa*						
Burundi	8.7(6.1–11)	27.7(15–61)	19.1(16–22)	9.3(7.6–11)	**—**	**—**
Comoros	2.6(1.9–3.7)	27.0(14–59)	21.3(19–24)	8.4(7.1–9.7)	**—**	**—**
Djibouti	3.5(2.5–4.8)	33.9(15–55)	17.4(15–20)	5.4(4.4–6.7)	**—**	**—**
Eritrea	10.6(8.1–13)	21.2(11–49)	17.0(15–20)	4.6(3.6–5.9)	**—**	**—**
Ethiopia	5.2(3.8–6.6)	25.6(15–52)	24.3(21–28)	8.5(6.9–10)	9.7 (6.3–20.3)	13.5 (11.6–15.3)
Kenya	2.9(2.2–3.6)	21.8(14–38)	18.6(17–21)	4.9(4.3–5.5)	9.7 (7.9–18.0)	11.3 (3.4–19.1)
Madagascar	4.2(3.3–5.2)	20.6(15–36)	14.0(13–16)	4.8(4.3–5.4)	**—**	18.1 (**—**)
Malawi	4.1(3.1–5.3)	23.7(17–35)	20.1(17–23)	6.4(5.5–7.7)	15.1 (13.3–18.3)	17.5 (17.3–17.6)
Mozambique	5.0(3.7–6.5)	28.4(17–51)	27.1(24–31)	10.5(9.0–12)	16.2 (**—**)	15.4 (**—**)
Rwanda	3.3(2.2–4.8)	22.8(16–36)	19.3(17–22)	5.8(4.9–6.7)	**—**	**—**
Somalia	4.6(3.2–6.3)	30.1(15–64)	16.5(14–19)	9.5(7.8–12)	**—**	**—**
Sudan	2.7(2.0–3.5)	23.9(17–40)	20.1(17–23)	7.2(6.0–8.8)	14.9 (8.3–18.0)	5.7 (**—**)
Tanzania	4.1(3.3–5.0)	25.6(19–40)	18.0(16–20)	5.7(5.1–6.4)	14.2 (8.6–22.4)	8.3 (7.9–10.0)
Uganda	2.7(2.0–3.4)	24.8(19–36)	20.6(18–23)	5.9(5.1–6.8)	9.6 (6.4–37.1)	20.3 (**—**)
Zambia	2.9(2.2–3.8)	25.5(18–40)	17.2(15–19)	8.5(7.4–9.6)	**—**	**—**
*Southern Africa*						
Botswana	5.1(3.6–6.8)	16.2 (9–37)	14.8(12–18)	3.4(2.4–4.6)	13.0 (**—**)	20.1 (**—**)
Lesotho	2.3(1.7–3.2)	25.2(14–54)	20.4(27–34)	8.1(6.7–9.7)	**—**	**—**
Namibia	1.3(1.0–1.8)	15.0(11–35)	18.5(16–21)	4.1(3.2–5.3)	**—**	**—**
South Africa	0.9(0.7–1.2)	20.4(14–31)	10.6(10–12)	3.4(3.1–3.8)	14.7 (13.8–16.3)	**—**
Swaziland	2.8(2.0–3.7)	18.2(11–36)	18.1(15–21)	5.0(3.8–6.6)	**—**	**—**
Zimbabwe	3.2(2.3–4.6)	20.0(13–35)	16.2(14–19)	5.9(4.5–7.6)	14.1 (6.0–20.3)	17.3 (**—**)
*West Africa*						
Benin	3.2(2.5–4.1)	24.3(17–41)	22.8(20–26)	5.8(4.8–6.9)	15.7 (10.0–17.8)	**—**
Burkina Faso	3.4(2.8–4.1)	26.2(19–40)	24.8(21–30)	13.1(10–16.5)	12.2 (—)	**—**
Cameroon*	5.2(4.0–6.7)	25.6(13–54)	25.2(22–29)	7.8(5.9–9.6)	16.4 (9.6–20.3)	20.3 (—)
Cape Verde	1.3(0.9–1.7)	15.6 (8–34)	10.8(9.0–13)	3.0(2.4–3.8)	8.2 (—)	13.4 (—)
Chad*	5.9(4.8–7.1)	29.2(14–64)	30.8(27–36)	13.7(11–16)	**—**	**—**
Côte d'Ivoire	4.4(3.3–5.8)	27.4(14–45)	26.2(22–30)	10.3(7.9–13)	10.6 (—)	**—**
The Gambia	2.7(1.9–3.5)	26.0(14–53)	22.4(19–26)	7.3(5.6–9.4)	18.6 (13.3–23.9)	21.4 (12.3–30.5)
Ghana	3.2(2.4–4.0)	22.0(14–37)	19.8(17–22)	4.7(4.0–5.6)	16.4 (13.3–20.3)	14.1 (—)
Guinea	6.5(5.1–7.9)	23.8(16–48)	28.5(25–33)	10.3(8.4–13)	**—**	**—**
Guinea-Bissau	8.2(6.3–10)	29.6(16–62)	30.9(26–36)	13.8(11–17)	13.3 (11.8–14.7)	**—**
Liberia	8.8(7.1–10)	26.9(14–56)	23.5(21–26)	7.6(6.5–9.0)	**—**	**—**
Mali	4.1(3.2–5.1)	23.2(18–42)	32.7(27–38)	12.1(9.7–15)	18.6 (—)	5.3 (—)
Mauritania	5.4(4–7.0)	27.4(17–51)	24.7(21–29)	6.3(4.8–8)	**—**	**—**
Niger	5.1(4.1–6.2)	22.9(17–41)	20.0(17–24)	11.6(9.6–14)	**—**	**—**
Nigeria	4.7(3.8–5.6)	41.7(25–72)	27.5(24–32)	9.9(8.3–11)	12.2 (5.5–29)	13.5 (10.6–19.4)
Sao Tome and Principe	2.6(3.3–3.3)	21.0(11–48)	16.7(15–19)	4.1(3.5–4.7)	**—**	**—**
Senegal	3.6(2.7–4.5)	33.8(27–50)	18.8(16–22)	6.6(5.3–7.9)	10.3 (9.5–18.8)	**—**
Sierra Leone	6.0(4.7–7.4)	30.0(16–66)	26.3(23–30)	9.0(7.5–11)	**—**	**—**
Togo	3.9(2.6–5.5)	25.0(13–54)	26.7(23–31)	6.9(5.6–8.7)	**—**	11.1 (—)

¥ Maternal and neonatal deaths were expressed as a fraction of total births by multiplying the maternal mortality ratio (maternal deaths/live births) and the neonatal mortality ratio (neonatal deaths/live birth) by 1 minus the stillbirth rate [44].

† For % LBW and % <37 wks, the median and range for all studies performed in the specified country are presented.

* Note –UN Data classifies Cameroon and Chad as falling within the Middle Africa sub-region rather than the West Africa sub-region. However, in this table these countries are kept within the West Africa sub-region to maintain congruity with global burden of disease publications.

## Discussion

We present a comprehensive review of pregnancy outcomes in Sub-Saharan Africa intended to inform safety assessments of current and future clinical trials conducted in pregnant women or neonates. The observation-time model of maternal and neonatal mortality accounts for the logistical realities of variation in gestational age at recruitment, time to delivery, and follow-up time. This model yields lower mortality estimates than multiplying mortality rates by cumulative live births, especially if a safety evaluation is conducted immediately after a cluster of deliveries. Overall, our review noted that the incidences of maternal and neonatal mortality, severe acute maternal morbidity, stillbirths, and major congenital malformations are highest in West Africa, the least developed sub-region of the continent. However, relatively few detailed published studies of severe acute maternal morbidity and congenital malformations in Sub-Saharan Africa exist, especially in the community setting.

Of all adverse pregnancy outcomes, the incidences of SAMM and MCM were the most variable across sub-regions. Studies with more stringent organ failure/management-based definitions found lower incidences of SAMM. Many studies of major congenital malformations were not included because malformations among live- and stillborn infants could not be separated. The limited time frame and diagnostic tools to detect congenital malformations in many studies may have led to systematic underreporting of specific types of malformations not readily apparent by physical examination at birth, such as many cardiovascular anomalies.

This compilation of pregnancy outcomes relevant to a maternal immunization clinical trial is consistent with the few other published summaries of pregnancy outcomes in Sub-Saharan Africa. Kaye et al [40] present a novel and pioneering review of causes of SAMM and case-fatality ratios. Although our review provides corroborating results, it also advances the field by providing several notable enhancements as we update the literature search, focus more explicitly on the background incidence of SAMM, and apply more stringent requirements for the catchment population. Careful interpretation is necessary to compare background rates of SAMM with those observed in a trial, as superior antenatal surveillance may decrease SAMM but better management of emergencies could prevent deaths, thereby elevating the rate of near-miss cases.

Our systematic review of LBW and prematurity does not yield nationally representative populations as are sought out in Demographic and Health Surveys and Multiple Indicator Cluster Surveys [41], [42]. Nevertheless, it allows designers of clinical trials to tailor expected background rates to recruitment sites by geographic and institutional setting. Despite this different approach, importantly, our overall estimates of the incidence of LBW are comparable to UNICEF values for sub-Saharan Africa overall (14%), Eastern and Southern Africa (14%), and West and Central Africa (13%) [43].

We recognize that our study has several limitations. First, we do not address differences in neonatal and maternal mortality rates among sub-populations within an individual country or across time that arise from geographic, socioeconomic, educational, and health access diversity. Thus, a DSMB should use its expertise and the best available data to select the most locally appropriate background rates for anticipated adverse event calculations. Second, pregnant women under the active surveillance of a clinical trial tend to have better access to medical care and therefore superior pregnancy outcomes relative to the general population. As in all mathematical models and systematic reviews, we are limited by the quality of the data informing our calculations. Many countries in Sub-Saharan Africa contribute incomplete vital registration data to systematic analyses of maternal mortality, stillbirths, and neonatal mortality [3], [44], [45] on which our calculations are based. Additionally, while our search included English, French, and Portuguese language publications, it ignores publications from sub-Saharan African countries in journals not included in MEDLINE.

The global public health community awaits with great anticipation the results of several ongoing clinical trials in developing countries that explore whether maternal immunization improves pregnancy outcomes and enhances young infant survival. In some of these trials, investigators have chosen to avoid the use of pure placebo in the control group, instead providing a licensed vaccine with an independent potential benefit that does not influence the primary outcome. For example, in an ongoing clinical trial in Mali assessing the effectiveness of maternal influenza immunization against influenza in mothers and their infants, the pregnant women randomized to the control group receive quadrivalent meningococcal conjugate vaccine (NCT01430689). In such situations where for ethical reasons the study design does not include a true placebo group, reliable background rates of adverse pregnancy outcomes are invaluable to help distinguish between vaccine benefits and safety concerns. Further studies that clarify locally relevant, population-based background rates of adverse pregnancy outcomes will improve safety assessment of maternal interventions.

## Supporting Information

Table S1 **Meta-analysis of the proportion of maternal deaths that occur before delivery, during labor and delivery or within 24 hours, and 24 hours to 42 days post-partum.**
(DOCX)

Table S2 **The incidence of low birth weight and prematurity in 104 studies in Sub-Saharan Africa published between 1992 and 2011.**
(DOCX)
