# Supplementary material for: Background Rates of Adverse Pregnancy Outcomes for Assessing the Safety of Maternal Vaccine Trials in Sub-Saharan Africa
Source: PLoS One. 2012 Oct 4;7(10):e46638. doi: 10.1371/journal.pone.0046638 (PMC3464282; doi:10.1371/journal.pone.0046638)
Supplement: Table S1 — Meta-analysis of the proportion of maternal deaths that occur before delivery, during labor and delivery or within 24 hours, and 24 hours to 42 days post-partum. (DOCX) [file pone.0046638.s001.docx]

**Supplement to:**

**Background rates of adverse pregnancy outcomes for assessing the safety of maternal vaccine trials in Sub-Saharan Africa**

Authors: Lauren A.V. Orenstein^1,2^ B.S., Evan W. Orenstein^1,2^ B.S., Ibrahima Teguete^3^ M.D., Mamoudou Kodio^2^ Pharm D., Milagritos Tapia^2,4^ M.D., Samba O. Sow^2,4^ M.D., M.Sc., Myron M. Levine^4^ M.D., D.T.P.H.*

^1^Emory University School of Medicine, Atlanta, GA, United States

^2^Centre pour le Développement des Vaccins-Mali, Bamako, Mali

^3^Gabriel Touré Teaching Hospital, Department of Obstetrics and Gynecology, Bamako, Mali

^4^ Center for Vaccine Development, University of Maryland School of Medicine, Baltimore, MD 21201, USA

**Table S1:** Meta-analysis of the proportion of maternal deaths that occur before delivery, during labor and delivery or within 24 hours, and 24 hours to 42 days post-partum.

| **Study Author** | **Years** | **Country** | **Study design** | **Maternal deaths** | **Before Delivery n, (%)** | **During Delivery n, (%)** | **1-42 days post-partum n, (%)** |
| --- | --- | --- | --- | --- | --- | --- | --- |
| McDermott [[1](#_ENREF_1)] | 1987-1989 | Malawi | Prospective cohort | 15 | 3 (20) | 4 (27) | 8 (53) |
| Martey [2] | 1985-1989 | Ghana | Population survey | 44 | 10 (23) | 32 (73) | 2 (5) |
| Hoj [3] | 1996-1997 | Guinea-Bissau | Population survey | 111 | 20 (18) | 48 (43) | 43 (39) |
| Bouvier-Collé [4] | 1994-1996 | 6 countries | Prospective cohort | 52 | 4 (8) | 20 (38) | 28 (54) |
| Walraven [5] | 1993-1998 | Gambia | Population survey | 18 | 8 (44) | 6 (33) | 4 (22) |
| Cham [6] | 2002 | Gambia | Record review and verbal autopsy | 42 | 4 (10) | 17 (40) | 21 (50) |
| Greenwood [7] | 1982-1983 | Gambia | Prospective cohort | 14 | 4 (29) | 6 (42) | 4 (29) |
|  |  |  | *Heterogeneity (I^2*, *p-value)* | | *68%, 0.008* | *78%, <0.001* | *---^a^* |
|  |  |  | *Analysis* | | *Random effects* | *Random effects* | *---* |
|  |  |  | ***Mean outcome*** | | **18.6% (14 – 24)** | **44.9% (35 – 53)** | **36.5% (27 – 47)** |

^a^The proportion of maternal deaths in the post-partum period was taken to be 1 minus the proportions before and during delivery.

**References:**

1. McDermott JM, Slutsker L, Steketee RW, Wirima JJ, Breman JG, et al. (1996) Prospective assessment of mortality among a cohort of pregnant women in rural Malawi. Am J Trop Med Hyg 55: 66-70.

2. Martey JO, Djan JO, Twum S, Browne EN, Opoku SA (1994) Maternal mortality and related factors in Ejisu District, Ghana. East Afr Med J 71: 656-660.

3. Hoj L, Stensballe J, Aaby P (1999) Maternal mortality in Guinea-Bissau: the use of verbal autopsy in a multi-ethnic population. Int J Epidemiol 28: 70-76.

4. Bouvier-Colle MH, Ouedraogo C, Dumont A, Vangeenderhuysen C, Salanave B, et al. (2001) Maternal mortality in West Africa. Rates, causes and substandard care from a prospective survey. Acta Obstet Gynecol Scand 80: 113-119.

5. Walraven G, Telfer M, Rowley J, Ronsmans C (2000) Maternal mortality in rural Gambia: levels, causes and contributing factors. Bull World Health Organ 78: 603-613.

6. Cham M, Vangen S, Sundby J (2007) Maternal deaths in rural Gambia. Glob Public Health 2: 359-372.

7. Greenwood AM, Greenwood BM, Bradley AK, Williams K, Shenton FC, et al. (1987) A prospective survey of the outcome of pregnancy in a rural area of the Gambia. Bull World Health Organ 65: 635-643.
