# Supplementary material for: Background Rates of Adverse Pregnancy Outcomes for Assessing the Safety of Maternal Vaccine Trials in Sub-Saharan Africa
Source: PLoS One. 2012 Oct 4;7(10):e46638. doi: 10.1371/journal.pone.0046638 (PMC3464282; doi:10.1371/journal.pone.0046638)
Supplement: Table S2 — The incidence of low birth weight and prematurity in 104 studies in Sub-Saharan Africa published between 1992 and 2011. (DOCX) [file pone.0046638.s002.docx]

**Supplement to:**

**Background rates of adverse pregnancy outcomes for assessing the safety of maternal vaccine trials in Sub-Saharan Africa**

Authors: Lauren A.V. Orenstein^1,2^ B.S., Evan W. Orenstein^1,2^ B.S., Ibrahima Teguete^3^ M.D., Mamoudou Kodio^2^ Pharm D., Milagritos Tapia^2,4^ M.D., Samba O. Sow^2,4^ M.D., M.Sc., Myron M. Levine^4^ M.D., D.T.P.H.*

^1^Emory University School of Medicine, Atlanta, GA, United States

^2^Centre pour le Développement des Vaccins-Mali, Bamako, Mali

^3^Gabriel Touré Teaching Hospital, Department of Obstetrics and Gynecology, Bamako, Mali

^4^ Center for Vaccine Development, University of Maryland School of Medicine, Baltimore, MD 21201, USA

**Table S2:** The incidence of low birth weight and prematurity in 104 studies in Sub-Saharan Africa published between 1992 and 2011.

| **sdf Study** | **Country** | **Years** | **Design^†^** | **Setting** | **N*** | **Mean Birth Weight (SD)** | **<2.5 kg (%)** | **<37 wks (%)** | **SGA (%)** | **Method of GA Estimation** | **SGA Definition** |
| --- | --- | --- | --- | --- | --- | --- | --- | --- | --- | --- | --- |
| *Central* | | | | | | | | | | | |
| Pambou [1] | Congo (Brazza) | 1994 | Record review | Urban hospital | 5109 TB^†^ | --- | --- | 16.7 | --- | LMP, US, or Ballard/Farr exam | --- |
| Mabiala-Babela [2] | Congo (Brazza) | 2005 | Record review | Urban hospitals | 3944 LB | --- | 12.4 | --- | --- | --- | --- |
| Mugisho [3] | DR Congo | 1980-1998 | Record review | Rural hospital | 13042 TB^†^ | --- | 18.0 | --- | --- | --- | --- |
| Newby [4] | DR Congo | 1989-1994 | Record review | Rural hospital | 4942 TB^†^ | 2669.0 (---) | 34.2 | --- | --- | --- | --- |
| Kyamusugulwa [5] | DR Congo | 2003-2004 | Perinatal survey | Rural hospitals | 938 TB^†^ | --- | 22.0 | --- | --- | --- | --- |
| Engmann [6] | DR Congo | 2005-2007 | Prospective cohort | Rural clinics | 7932 LB^†^ | 3071.0 (537.8) | 10.5 | 2.3 | --- | LMP | --- |
| Ramharter [7] | Gabon | 2003-2006 | Perinatal survey | Urban and semi-urban hospitals | 1173 TB | 3055.6 (507.7) | 10.7 | 21.3 | --- | LMP | --- |
| Kurth [8] | Gabon | 2005-2006 | Perinatal survey | Urban hospitals | 775 LB | 3076.6 (500.2) | 10.3 | 19.1 | 4.7 | LMP | Term LBW |
| *East* | | | | | | | | | | | |
| Gessessew [9] | Ethiopia | 1993-2003 | Record review | Semi-urban hospital | 7150 TB | 3108.6 (915.1) | 11.2 | --- | --- | --- | --- |
| Teshome [10] | Ethiopia | 1995-1996 | Perinatal survey | Urban hospital | 804 TB^†^ | 2978 (---) | 15.4 | 15.3 | --- | LMP | --- |
| Feleke [11] | Ethiopia | 1996-1997 | Perinatal survey | Urban hospitals | 4010 LB | 3065 (465) | 9.1 | 11.6 | 6.4 | LMP | Term LBW |
| Gebremariam [12] | Ethiopia | 1999-2000 | Perinatal survey | Urban hospital | 1441 LB^†^ | --- | 10.2 | --- | --- | --- | --- |
| Tema [13] | Ethiopia | 2002-2003 | Perinatal survey | Urban/rural clinics and hospital | 595 LB | --- | 20.3 | --- | --- | --- | --- |
| Nekatibeb [14] | Ethiopia | 2003-2005 | Record review | Semi-urban hospital | 1832 LB | 3147 (---) | 8.6 | --- | --- | --- | --- |
| Belete [15] | Ethiopia | 2004 | Perinatal survey | Urban hospitals | 320 TB | 3152 (494) | 6.3 | --- | --- | --- | --- |
| Hanlon [16] | Ethiopia | 2005-2006 | Prospective cohort | Rural community | 521 LB | 3005.9 (382.7) | 7.3 | --- | --- | --- | --- |
| Allen [17] | Kenya | 1981-1992 | Prospective cohort | Rural community | 140 LB^†^ | 3150 (400) | 10.0 | --- | --- | --- | --- |
| Aidoo [18] | Kenya | 1992-1994 | Prospective cohort | Rural communities | 990 LB | 3082.6 (457.9) | 8.6 | 3.4 | 20.1 | Dubowitz exam | Not defined |
| Shulman [19] | Kenya | 1996-1997 | Perinatal survey | Rural hospital | 820 LB | 2900 (510) | 18.0 | --- | --- | --- | --- |
| Temmerman [20] | Kenya | 1997-1998 | Perinatal survey | Urban hospital | 12414 TB^†^ | --- | 8.8 | --- | --- | --- | --- |
| Andriamady [21] | Madagascar | 1997 | Record review | Urban hospital | 7717 TB | --- | --- | 18.1 | --- | Dubowitz exam | --- |
| Mwanyumba [22] | Kenya | 1999 | Perinatal survey | Urban hospital | 627 LB | 2968.6 (535.5) | 12.4 | --- | 6.5 | Dubowitz exam | Term LBW |
| van Eijk [23] | Kenya | 1999-2000 | Perinatal survey | Urban hospital | 2114 LB | 3091.9 (484.4) | 9.4 | --- | --- | --- | --- |
| English [24] | Kenya | 1999-2001 | Perinatal survey | Rural hospital | 2857 LB | 2949.1 (507.4) | 16.5 | --- | --- | --- | --- |
| Brown [25] | Kenya | 2004-2005 | Prospective cohort | Rural communities | 1340 LB^†^ | --- | 7.9 | --- | --- | --- | --- |
| Alusala [26] | Kenya | --- | Prospective cohort | Urban and rural clinic | 578 TB | 3107 (480.8) | 8.5 | --- | --- | --- | --- |
| Kavoo-Linge [27] | Kenya | --- | Perinatal survey | Semi-urban hospital | 1876 TB^†^ | --- | 12.4 | 19.1^‡^ | --- | --- | --- |
| Kalanda [28] | Malawi | 1993-1994 | Prospective cohort | Rural hospitals | 1414 LB | 2938.6 (449.3) | 14.9 | 17.3 | 20.3 | Modified Ballard [29] | <10^th^ percentile [30] |
| Kulmala [31] | Malawi | 1995-1996 | Prospective cohort | Rural clinic | 476 LB | 3099.5 (505.9) | 15.1 | --- | --- | --- | --- |
| Vaahtera [32] | Malawi | 1995-1996 | Prospective cohort | Rural clinic | 480 LB^†^ | 3069 (523) | 18.3 | --- | --- | --- | --- |
| Rogerson [33] | Malawi | 1997-1999 | Perinatal survey | Urban hospital | 1044 LB | --- | 15.2 | --- | --- | --- | --- |
| van den Broek [34] | Malawi | --- | Prospective cohort | Rural clinic and hospital | 218 TB^†^ | --- | 13.3 | 17.6^‡^ | --- | US <24 wks | --- |
| Osman [35] | Mozambique | 1993-1995 | Prospective cohort | Semi-urban clinics | 821 TB | 2910 (---) | 16.2 | 15.4 | 9.7 | US < 21 wks | Not defined |
| Adam [36] | Sudan | 2006-2007 | Perinatal survey | Rural hospital | 524 TB | 3053.6 (559.3) | 15.3 | --- | --- | --- | --- |
| Haggaz [37] | Sudan | 2008 | Perinatal survey | Urban hospital | 430 LB | 2985.9 (574.8) | 14.9 | --- | --- | --- | --- |
| Hassan [38] | Sudan | 2008 | Perinatal survey | Urban hospital | 2076 TB | 3040.6 (573.3) | 12.5 | --- | --- | --- | --- |
| Aziz [39] | Sudan | --- | Perinatal survey | Rural community | 1794 LB^†^ | --- | 18.0 | --- | --- | --- | --- |
| Elshibly [40] | Sudan | --- | Perinatal survey | Urban hospital | 1000 LB | 3131.7 (538.9) | 8.3 | 5.7 | --- | LMP | --- |
| Wort [41] | Tanzania | 1990-1999 | Record review | Rural hospital | 11903 LB | 2997.2 (944.9) | 10.6 | --- | --- | --- | -- |
| Menendez [42] | Tanzania | 1994-1995 | Perinatal survey | Semi-urban hospital | 1177 LB | 2818.7 (429.0) | 17.6 | 7.9 | 13.4 | Dubowitz exam | Term LBW |
| Hinderaker [43] | Tanzania | 1995-1996 | Perinatal survey | Rural hospital | 774 TB^†^ | --- | 9.7 | --- | --- | --- | --- |
| Jamieson [44] | Tanzania | 1997-1998 | Record review | Refugee camp | 617 LB^†^ | --- | 22.4 | --- | --- | --- | --- |
| Wort [45] | Tanzania | 1997-2001 | Record review | Rural hospitals and clinics | 9839 LB | 2935.7 (491.0) | 12.2 | --- | --- | --- | --- |
| Kidanto [46] | Tanzania | 1999-2003 | Perinatal survey | Urban hospital | 67972 LB | --- | 19.0 | --- | --- | --- | --- |
| Muganyizi [47] | Tanzania | 1999-2005 | Record review | Urban hospital | 91699 TB | --- | 16.2 | --- | --- | --- | --- |
| Habib [48] | Tanzania | 1999-2006 | Record review | Urban hospital | 14444 TB | 3191 (453) | --- | 10.0 | --- | LMP | --- |
| Mmbaga [49] | Tanzania | 2000-2008 | Perinatal survey | Urban hospital | 21146 LB | 3090 (544) | 8.6 | --- | --- | --- | --- |
| Siza [50] | Tanzania | 2001 | Record review | Semi-urban hospital | 3464 TB^†^ | --- | 13.6 | --- | --- | --- | --- |
| Wort [51] | Tanzania | 2001-2002 | Perinatal survey | Rural hospital | 1684 LB | 2892.3 (464.6) | 15.7 | --- | --- | --- | --- |
| Mmbando [52] | Tanzania | 2004-2005 | Record review | Rural/semi-urban clinics | 1458 TB | 3010 (572.8) | 9.9 | --- | --- | --- | --- |
| Marchant [53] | Tanzania | 2009 | Perinatal survey | Rural hospital | 529 LB^†^ | 2900 (400) | 14.7 | 8.3 | --- | Eregie exam [54] | --- |
| Azayo [55] | Tanzania | --- | Perinatal survey | Urban tertiary care hospital | 150 LB^†^ | 3200 (---) | 12.0 | --- | --- | --- | --- |
| Ndyomugyenyi [56] | Uganda | 1997-1998 | Record review | Rural hospitals | 5729 LB | 3072.0 (470.1) | 9.9 | --- | --- | --- | --- |
| Kasumba [57] | Uganda | 1998 | Perinatal survey | Urban hospital | 514 LB^†^ | 3059.0 (548.3) | 9.3 | --- | --- | --- | --- |
| Kaye [58] | Uganda | 2004-2005 | Prospective cohort | Urban hospital | 612 LB | 2782.5 (626.0) | 37.1 | 20.3 | 18.8 | LMP, US, or exam | Not defined |
| Byaruhanga [59] | Uganda | --- | Perinatal survey | Urban hospital | 300 LB^†^ | 3218 (496) | 6.4 | --- | --- | --- | --- |
| *Southern* | | | | | | | | | | | |
| Letamo [60] | Botswana | 1990-1995 | Record review | Urban and rural hospitals | 6735 TB^†^ | 3055.0 (563.4) | 13.0 | --- | --- | --- | --- |
| Parekh [61] | Botswana | 2007-2010 | Record review | Urban/semi-urban/rural hospitals | 16219 LB^†^ | --- | --- | 20.1 | 11.3 | LMP | Botswana [90] |
| Louw [62] | South Africa | 1989-1991 | Record review | Urban/semi-urban hospitals/clinics | 367092 LB^†^ | --- | 14.7 | --- | --- | --- | --- |
| Woods [63] | South Africa | 1993 | Record review | Regional database | 20668 TB^¥^ | --- | 17.2 | --- | --- | --- | --- |
| Pattinson [64] | South Africa | 1999-2002 | Record review | Nationwide database | 227504 LB^†^ | --- | 16.3 | --- | --- | --- | --- |
| Hoque [65] | South Africa | 2005 | Record review | Rural hospital | 7836 TB^†^ | 2985.4 | 13.8 | --- | --- | --- | --- |
| Le Bacq [66] | Zimbabwe | 1990-1991 | Perinatal survey | Urban hospital and rural communities | 684 TB^†^ | --- | 9.4 | --- | --- | --- | --- |
| Kambarami [67] | Zimbabwe | 1994 | Record review | Rural communities | 419 LB^†^ | --- | 14.3 | --- | --- | --- | --- |
| Majoko [68] | Zimbabwe | 1995-1998 | Prospective cohort | Rural clinics | 10497 TB^†^ | 3154.6 (490.8) | 6.0 | --- | --- | --- | --- |
| Galvan [69] | Zimbabwe | 1996-1998 | Perinatal survey | Urban hospital | 3855 TB | 2961.1 (573.3) | 15.6 | --- | --- | --- | --- |
| Feresu [70] | Zimbabwe | 1997-1998 | Record review | Urban hospital | 16164 LB^†^ | 2879 | 20.3 | 17.3 | 9.2 | LMP | Term LBW |
| Ogbonna [71] | Zimbabwe | 1998-1999 | Perinatal survey | Urban hospital | 498 TB | --- | 14.1 | --- | --- | --- | --- |
| Ticconi [72] | Zimbabwe | 2000-2001 | Record review | Rural hospital | 1705 TB^†^ | --- | 12.9 | --- | --- | --- | --- |
| *West* | | | | | | | | | | | |
| Fourn [73] | Benin | 1991 | Prospective cohort | Urban clinics | 4113 LB^†^ | --- | 17.8 | --- | --- | --- | --- |
| Denoeud [74] | Benin | 2004-2005 | Perinatal survey | Semi-urban clinics | 1087 LB | 2850 (491.2) | 15.7 | --- | --- | --- | --- |
| Le Port [75] | Benin | 2007-2008 | Perinatal survey | Rural clinics | 588 LB | 2979.0 (402.2) | 10.0 | --- | --- | --- | --- |
| Sirima [76] | Burkina Faso | 2004 | Prospective cohort | Semi-urban clinics | 1133 LB | 2981 (438) | 12.2 | --- | ---- | --- | --- |
| Tako [77] | Cameroon | 1996-2001 | Perinatal survey | Urban hospital | 1708 TB | 3002.1 (732.0) | 16.4 | 20.3 | --- | LMP, exam at birth | --- |
| Salihu [78] | Cameroon | 1997-1998 | Perinatal survey | Semi-urban hospital | 232 LB^†^ | --- | 20.3 | --- | --- | --- | --- |
| Akum [79] | Cameroon | 1999-2001 | Perinatal survey | Semi-urban clinic | 749 LB | 3140 (480) | 9.6 | --- | --- | --- | --- |
| Wessel [80] | Cape Verde | 1991-1992 | Prospective cohort | Urban and rural clinics | 330 TB | 3169.5 (---) | 8.2 | 13.4 | --- | LMP | --- |
| Vanga-Bosson [81] | Côte d’Ivoire | 2008 | Perinatal survey | Urban and semi-urban clinics | 1945 LB | --- | 10.6 | --- | --- | --- | --- |
| Rayco-Solon [82] | Gambia | 1976-2003 | Retrospective cohort | Rural communities | 2472 LB^†^ | --- | 13.3 | 12.3 | 25.1 | Dubowitz exam | <10th percentile [51] |
| Okoko [83] | Gambia | 1997 | Perinatal survey | Rural hospital | 213 LB | --- | 23.9 | 30.5 | 8.5 | Dubowitz exam | Term LBW |
| Klufio [84] | Ghana | 1994 | Perinatal survey | Urban hospital | 866 LB | 3070 (616) | 13.3 | 14.1 | --- | US <26 wks, LMP | --- |
| Shuaib [85] | Ghana | 2006 | Perinatal survey | Urban hospitals | 755 TB | --- | 20.3 | --- | --- | --- | --- |
| Oduro [86] | Ghana | 2006-2007 | Prospective cohort | Rural communities | 2182 LB | 2884.5 (486.6) | 16.4 | --- | --- | --- | --- |
| Roth [87] | Guinea-Bissau | 1989-1999 | Record review | Semi-urban hospital | 7138 LB^†^ | --- | 11.8 | --- | --- | --- | --- |
| Sodemann [88] | Guinea-Bissau | 1997-2002 | Record review | Semi-urban hospital | 2926 LB^†^ | --- | 14.7 | --- | --- | --- | --- |
| Kayentao [89] | Mali | 1998-1999 | Perinatal survey | Rural clinics | 453 TB | --- | 18.6 | 5.3 | --- | Ballard exam | --- |
| Famanta [90] | Mali | 2009 | Perinatal survey | Urban clinic | 372 TB^†^ | --- | 12.1 | --- | --- | --- | --- |
| Onah [91] | Nigeria | 1991-2000 | Record review | Urban hospital | 496 TB | 3200 (600) | --- | 10.6 | --- | LMP, US | --- |
| Egwunyenga [92] | Nigeria | 1994-1995 | Perinatal survey | Urban and rural hospitals | 1905 TB^†^ | --- | 11.3 | --- | --- | --- | --- |
| Lawoyin [93] | Nigeria | 1996-1998 | Prospective cohort | Rural community | 526 LB^†^ | --- | 24.1 | --- | --- | --- | --- |
| Ezeaka [94] | Nigeria | 1997 | Perinatal survey | Urban hospitals | 788 LB | 3046 (656) | 17.3 | --- | --- | --- | --- |
| Ozumba [95] | Nigeria | 1997-1999 | Record review | Urban hospital | 7160 LB^†^ | --- | 5.5 | --- | --- | --- | --- |
| Onah [96] | Nigeria | 1998 | Record review | Urban hospital | 851 LB | 3144 (640) | 10.1 | 10.9 | 6.2 | LMP, US | Not defined |
| Onyiriuka [97] | Nigeria | 1999-2001 | Perinatal survey | Urban hospital | 4075 LB^†^ | --- | 8.1 | --- | --- | --- | --- |
| Ibekwe [98] | Nigeria | 2001-2003 | Record review | Rural hospital | 1684 TB^†^ | --- | 16.1 | --- | --- | --- | --- |
| Ekure [99] | Nigeria | 2002 | Perinatal survey | Urban hospital | 560 LB^†^ | 3100 | 13.0 | --- | --- | --- | --- |
| Falade [100] | Nigeria | 2003-2004 | Perinatal survey | Urban hospital | 980 LB | 3153.6 (490) | 6.5 | --- | --- | --- | --- |
| Fawole [101] | Nigeria | 2004-2005 | Record review | Urban hospitals and clinics | 9208 TB^†^ | --- | 10.1 | --- | --- | --- | --- |
| Aribodor [102] | Nigeria | 2005-2006 | Perinatal survey | Urban and rural communities | 500 TB | 2963.0 (---) | 29.0 | --- | --- | --- | --- |
| Sotimehin [103] | Nigeria | 2004-2005 | Perinatal survey | Urban hospital | 192 LB^†^ | 2910.3 (623.4) | 27.1 | 16.1 | 14.1 | LMP, Dubowitz exam | [104] |
| Tongo [105] | Nigeria | 2007-2008 | Perinatal survey | Urban hospital | 796 TB^†^ | 3020 (560) | 9.0 | 19.4 | --- | LMP, US, Ballard exam | --- |
| Sule-Odu [106] | Nigeria | --- | Perinatal survey | Urban hospital | 564 TB | 3035.2 (414.9) | 15.6 | --- | --- | --- | --- |
| Akanbi [107] | Nigeria | --- | Prospective cohort | Urban clinic | 254 TB^†^ | 2833.5 (730.7) | 17.7 | --- | --- | --- | --- |
| Akpala [108] | Nigeria | --- | Prospective cohort | Rural community | 1479 TB | --- | 8.8 | --- | --- | --- | --- |
| Camara [109] | Senegal | 1992-1993 | Record review | Semi-urban clinic | 7527 LB^†^ | --- | 10.3 | --- | --- | --- | --- |
| Olliaro [110] | Senegal | 2000-2007 | Record review | Rural clinic | 904 TB^†^ | --- | 9.5 | --- | --- | --- | --- |
| Gueye [111] | Senegal | 2006 | Perinatal survey | Urban hospital | 1152 TB | --- | 18.8 | --- | --- | --- | --- |
| Balaka [112] | Togo | --- | Perinatal survey | Urban hospital | 1672 LB^†^ | --- | --- | 11.1 | --- | US<12 wks, LMP, Dubowitz/Farr exam | --- |

**^†^** We classified studies into three designs: cohort studies in which pregnant women were recruited before delivery and pregnancy outcome was determined regardless of delivery site, perinatal surveys in which pregnant women were recruited in labor or immediately after delivery, and record reviews.
***** When the denominators for LBW and gestational age differed, we only included the denominator used for LBW.
^‡^ For Kavoo-Linge et al [27], the denominator for the % <37 wks was 1807 live births; For Van den Broek et al [34], the denominator for prematurity was 409 live births.
^¥^ For Woods et al [63], the % <2.5kg is expressed per live births, but the denominator was only given in total births.

**References**

1. Pambou O, Ntsika-Kaya P, Ekoundzola JR, Mayanda F (2006) [Preterm births at Brazzaville University Hospital]. Sante 16: 185-189.

2. Mabiala-Babela JR, Matingou VC, Senga P (2007) [Risk factors for low birth weight in Brazzaville, Congo]. J Gynecol Obstet Biol Reprod (Paris) 36: 795-798.

3. Mugisho E, Dramaix M, Porignon D, Musubao E, Hennart P (2002) [Analysis of data routinely collected in the maternity ward of Rutshuru in the Democratic Republic of the Congo between 1980 and 1998. II. New-born deaths and low birth weights]. Sante 12: 252-255.

4. Newby R, Lovel H (1995) Low birthweight at Kasaji Hospital, Zaire. Lancet 346: 1493-1494.

5. Milabyo Kyamusugulwa P (2006) [Low birth weight in Maniema (Democratic Republic of Congo)]. Sante 16: 103-107.

6. Engmann C, Matendo R, Kinoshita R, Ditekemena J, Moore J, et al. (2009) Stillbirth and early neonatal mortality in rural Central Africa. Int J Gynaecol Obstet 105: 112-117.

7. Ramharter M, Schuster K, Bouyou-Akotet MK, Adegnika AA, Schmits K, et al. (2007) Malaria in pregnancy before and after the implementation of a national IPTp program in Gabon. Am J Trop Med Hyg 77: 418-422.

8. Kurth F, Belard S, Mombo-Ngoma G, Schuster K, Adegnika AA, et al. (2010) Adolescence as risk factor for adverse pregnancy outcome in Central Africa--a cross-sectional study. PLoS One 5: e14367.

9. Gessessew A (2007) Twin deliveries in a zonal hospital: ten years retrospective study. Ethiop Med J 45: 55-59.

10. Teshome D, Telahun T, Solomon D, Abdulhamid I (2006) A study on birth weight in a teaching-referral hospital, Gondar, Ethiopia. Cent Afr J Med 52: 8-11.

11. Feleke Y, Enquoselassie F (1999) Maternal age, parity and gestational age on the size of the newborn in Addis Ababa. East Afr Med J 76: 468-471.

12. Gebremariam A (2005) Factors predisposing to low birth weight in Jimma Hospital south western Ethiopia. East Afr Med J 82: 554-558.

13. Tema T (2006) Prevalence and determinants of low birth weight in Jimma Zone, Southwest Ethiopia. East Afr Med J 83: 366-371.

14. Nekatibeb G, A GM (2007) Analysis of birth weight in Metu Karl hospital: South West Ethiopia. Ethiop Med J 45: 195-202.

15. Belete W, Gaym A (2008) Clinical estimation of fetal weight in low resource settings: comparison of Johnson's formula and the palpation method. Ethiop Med J 46: 37-46.

16. Hanlon C, Medhin G, Alem A, Tesfaye F, Lakew Z, et al. (2009) Impact of antenatal common mental disorders upon perinatal outcomes in Ethiopia: the P-MaMiE population-based cohort study. Trop Med Int Health 14: 156-166.

17. Allen LH, Lung'aho MS, Shaheen M, Harrison GG, Neumann C, et al. (1994) Maternal body mass index and pregnancy outcome in the Nutrition Collaborative Research Support Program. Eur J Clin Nutr 48 Suppl 3: S68-76; discussion S76-67.

18. Aidoo M, McElroy PD, Kolczak MS, Terlouw DJ, ter Kuile FO, et al. (2001) Tumor necrosis factor-alpha promoter variant 2 (TNF2) is associated with pre-term delivery, infant mortality, and malaria morbidity in western Kenya: Asembo Bay Cohort Project IX. Genet Epidemiol 21: 201-211.

19. Shulman CE, Marshall T, Dorman EK, Bulmer JN, Cutts F, et al. (2001) Malaria in pregnancy: adverse effects on haemoglobin levels and birthweight in primigravidae and multigravidae. Trop Med Int Health 6: 770-778.

20. Temmerman M, Gichangi P, Fonck K, Apers L, Claeys P, et al. (2000) Effect of a syphilis control programme on pregnancy outcome in Nairobi, Kenya. Sex Transm Infect 76: 117-121.

21. Andriamady RC, Rasamoelisoa JM, Rakotonoel H, Ravaonarivo H, Ranjalahy RJ, et al. (1999) [Premature deliveries at the maternity hospital of Befelatanana, Antananarivo in 1997]. Arch Inst Pasteur Madagascar 65: 93-95.

22. Mwanyumba F, Inion I, Gaillard P, Mandaliya K, Praet M, et al. (2003) Placental inflammation and perinatal outcome. Eur J Obstet Gynecol Reprod Biol 108: 164-170.

23. van Eijk AM, Ayisi JG, ter Kuile FO, Otieno JA, Misore AO, et al. (2004) Effectiveness of intermittent preventive treatment with sulphadoxine-pyrimethamine for control of malaria in pregnancy in western Kenya: a hospital-based study. Trop Med Int Health 9: 351-360.

24. English M, Muhoro A, Aluda M, Were S, Ross A, et al. (2003) Outcome of delivery and cause-specific mortality and severe morbidity in early infancy: a Kenyan District Hospital birth cohort. Am J Trop Med Hyg 69: 228-232.

25. Brown CA, Sohani SB, Khan K, Lilford R, Mukhwana W (2008) Antenatal care and perinatal outcomes in Kwale district, Kenya. BMC Pregnancy Childbirth 8: 2.

26. Alusala DN, Estambale BB (2009) Intermittent presumptive treatment of malaria to prevent low birth weight in newborns in a cohort of pregnant women from a malaria endemic area. East Afr Med J 86: 378-386.

27. Kavoo L, Rogo KO (1992) Factors influencing early perinatal mortality in a rural district hospital. East Afr Med J 69: 181-187.

28. Kalanda BF, Verhoeff FH, Chimsuku L, Harper G, Brabin BJ (2006) Adverse birth outcomes in a malarious area. Epidemiol Infect 134: 659-666.

29. Verhoeff FH, Milligan P, Brabin BJ, Mlanga S, Nakoma V (1997) Gestational age assessment by nurses in a developing country using the Ballard method, external criteria only. Ann Trop Paediatr 17: 333-342.

30. Williams RL, Creasy RK, Cunningham GC, Hawes WE, Norris FD, et al. (1982) Fetal growth and perinatal viability in California. Obstet Gynecol 59: 624-632.

31. Kulmala T, Vaahtera M, Ndekha M, Koivisto AM, Cullinan T, et al. (2000) The importance of preterm births for peri- and neonatal mortality in rural Malawi. Paediatr Perinat Epidemiol 14: 219-226.

32. Vaahtera M, Kulmala T, Ndekha M, Koivisto AM, Cullinan T, et al. (2000) Antenatal and perinatal predictors of infant mortality in rural Malawi. Arch Dis Child Fetal Neonatal Ed 82: F200-204.

33. Rogerson SJ, Chaluluka E, Kanjala M, Mkundika P, Mhango C, et al. (2000) Intermittent sulfadoxine-pyrimethamine in pregnancy: effectiveness against malaria morbidity in Blantyre, Malawi, in 1997-99. Trans R Soc Trop Med Hyg 94: 549-553.

34. van den Broek N, Ntonya C, Kayira E, White S, Neilson JP (2005) Preterm birth in rural Malawi: high incidence in ultrasound-dated population. Hum Reprod 20: 3235-3237.

35. Osman NB, Challis K, Cotiro M, Nordahl G, Bergstrom S (2000) Maternal and fetal characteristics in an obstetric cohort in Mozambique. Afr J Reprod Health 4: 110-119.

36. Adam I, Ismail MH, Nasr AM, Prins MH, Smits LJ (2009) Low birth weight, preterm birth and short interpregnancy interval in Sudan. J Matern Fetal Neonatal Med 22: 1068-1071.

37. Haggaz AD, Radi EA, Adam I (2010) Anaemia and low birthweight in western Sudan. Trans R Soc Trop Med Hyg 104: 234-236.

38. Hassan AA, Abubaker MS, Radi EA, Adam I (2009) Education, prenatal care, and poor perinatal outcome in Khartoum, Sudan. Int J Gynaecol Obstet 105: 66-67.

39. Aziz MI, Ahmed ME, Al Rahman NB (1996) Discovering the extent of low birth weight in rural Sudan. World Health Forum 17: 402-403.

40. Elshibly EM, Schmalisch G (2008) The effect of maternal anthropometric characteristics and social factors on gestational age and birth weight in Sudanese newborn infants. BMC Public Health 8: 244.

41. Wort UU, Hastings IM, Carlstedt A, Mutabingwa TK, Brabin BJ (2004) Impact of El Nino and malaria on birthweight in two areas of Tanzania with different malaria transmission patterns. Int J Epidemiol 33: 1311-1319.

42. Menendez C, Ordi J, Ismail MR, Ventura PJ, Aponte JJ, et al. (2000) The impact of placental malaria on gestational age and birth weight. J Infect Dis 181: 1740-1745.

43. Hinderaker SG, Olsen BE, Bergsjo PB, Gasheka P, Lie RT, et al. (2003) Perinatal mortality in northern rural Tanzania. J Health Popul Nutr 21: 8-17.

44. Jamieson DJ, Meikle SF, Hillis SD, Mtsuko D, Mawji S, et al. (2000) An evaluation of poor pregnancy outcomes among Burundian refugees in Tanzania. JAMA 283: 397-402.

45. Uddenfeldt Wort U, Warsame M, Brabin BJ (2008) Potential use of birthweight indicators in rural Tanzania for monitoring malaria control in pregnancy. Public Health 122: 923-932.

46. Kidanto HL, Massawe SN, Nystrom L, Lindmark G (2006) Analysis of perinatal mortality at a teaching hospital in Dar es Salaam, Tanzania, 1999-2003. Afr J Reprod Health 10: 72-80.

47. Muganyizi PS, Kidanto HL (2009) Impact of change in maternal age composition on the incidence of Caesarean section and low birth weight: analysis of delivery records at a tertiary hospital in Tanzania, 1999-2005. BMC Pregnancy Childbirth 9: 30.

48. Habib NA, Dalveit AK, Mlay J, Oneko O, Shao J, et al. (2008) Birthweight and perinatal mortality among singletons and twins in north-eastern Tanzania. Scand J Public Health 36: 761-768.

49. Mmbaga BT, Lie RT, Kibiki GS, Olomi R, Kvale G, et al. (2011) Transfer of newborns to neonatal care unit: a registry based study in Northern Tanzania. BMC Pregnancy Childbirth 11: 68.

50. Siza JE (2008) Risk factors associated with low birth weight of neonates among pregnant women attending a referral hospital in northern Tanzania. Tanzan J Health Res 10: 1-8.

51. Wort UU, Warsame M, Brabin BJ (2006) Birth outcomes in adolescent pregnancy in an area with intense malaria transmission in Tanzania. Acta Obstet Gynecol Scand 85: 949-954.

52. Mmbando BP, Cole-Lewis H, Sembuche S, Kamugisha ML, Theander T, et al. (2008) Risk factors for low birth-weight in areas with varying malaria transmission in Korogwe, Tanzania: implications for malaria control. Tanzan J Health Res 10: 137-143.

53. Marchant T, Jaribu J, Penfold S, Tanner M, Armstrong Schellenberg J (2010) Measuring newborn foot length to identify small babies in need of extra care: a cross sectional hospital based study with community follow-up in Tanzania. BMC Public Health 10: 624.

54. Eregie CO (2000) A new method for maturity determination in newborn infants. J Trop Pediatr 46: 140-144.

55. Azayo MM, Manji K, Kalokola F (2009) Lead levels in women at delivery at the Muhimbili National Hospital: a public health problem. J Trop Pediatr 55: 138-139.

56. Ndyomugyenyi R, Magnussen P (2001) Malaria morbidity, mortality and pregnancy outcome in areas with different levels of malaria transmission in Uganda: a hospital record-based study. Trans R Soc Trop Med Hyg 95: 463-468.

57. Kasumba IN, Nalunkuma AJ, Mujuzi G, Kitaka FS, Byaruhanga R, et al. (2000) Low birthweight associated with maternal anaemia and Plasmodium falciparum infection during pregnancy, in a peri-urban/urban area of low endemicity in Uganda. Ann Trop Med Parasitol 94: 7-13.

58. Kaye DK, Mirembe FM, Bantebya G, Johansson A, Ekstrom AM (2006) Domestic violence during pregnancy and risk of low birthweight and maternal complications: a prospective cohort study at Mulago Hospital, Uganda. Trop Med Int Health 11: 1576-1584.

59. Byaruhanga R, Bergstrom A, Okong P (2005) Neonatal hypothermia in Uganda: prevalence and risk factors. J Trop Pediatr 51: 212-215.

60. Letamo G, Majelantle RG (2001) Health implications of early childbearing on pregnancy outcome in Botswana: insights from the institutional records. Soc Sci Med 52: 45-52.

61. Parekh N, Ribaudo H, Souda S, Chen J, Mmalane M, et al. (2011) Risk factors for very preterm delivery and delivery of very-small-for-gestational-age infants among HIV-exposed and HIV-unexposed infants in Botswana. Int J Gynaecol Obstet 115: 20-25.

62. Louw HH, Khan MB, Woods DL, Power M, Thompson MC (1995) Perinatal mortality in the Cape Province, 1989-1991. S Afr Med J 85: 352-355.

63. Woods D, Khan M, Louw H (2001) A comparison of the perinatal mortality rates for infants weighing 500 g or more at birth and those weighing 1,000 g or more. S Afr Med J 91: 323-324.

64. Pattinson RC (2003) Why babies die--a perinatal care survey of South Africa, 2000-2002. S Afr Med J 93: 445-450.

65. Hoque M, Hoque S (2010) Comparison of perinatal and obstetrics outcomes among early adolescents, late adolescents and adult pregnant women from rural South Africa. East Afr J Public Health 7: 171-176.

66. Le Bacq F, Charimari L (1993) Birth weight patterns in a commercial farming area, rural area and urban area of Karoi District, Zimbabwe. J Trop Pediatr 39: 238-242.

67. Kambarami RA, Chirenje MZ, Rusakaniko S (2000) Perinatal practices in two rural districts of Zimbabwe: a community perspective. Cent Afr J Med 46: 96-100.

68. Majoko FM, Nystrom L, Munjanja SP, Mason E, Lindmark G (2004) Relation of parity to pregnancy outcome in a rural community in Zimbabwe. Afr J Reprod Health 8: 198-206.

69. Galvan J, Woelk GB, Mahomed K, Wagner N, Mudzamiri S, et al. (2001) Prenatal care utilization and foetal outcomes at Harare Maternity Hospital, Zimbabwe. Cent Afr J Med 47: 87-92.

70. Feresu SA, Harlow SD, Welch K, Gillespie BW (2004) Incidence of and socio-demographic risk factors for stillbirth, preterm birth and low birthweight among Zimbabwean women. Paediatr Perinat Epidemiol 18: 154-163.

71. Ogbonna C, Woelk GB, Ning Y, Mudzamiri S, Mahomed K, et al. (2007) Maternal mid-arm circumference and other anthropometric measures of adiposity in relation to infant birth size among Zimbabwean women. Acta Obstet Gynecol Scand 86: 26-32.

72. Ticconi C, Arpino C, Longo B, Mapfumo M (2005) Prevalence and risk factors for low birth weight in Northern Zimbabwe. Int J Gynaecol Obstet 88: 146-147.

73. Fourn L, Ducic S, Seguin L (1999) [Factors associated with low birth weight: a multivariate analysis]. Sante 9: 7-11.

74. Denoeud L, Fievet N, Aubouy A, Ayemonna P, Kiniffo R, et al. (2007) Is chloroquine chemoprophylaxis still effective to prevent low birth weight? Results of a study in Benin. Malar J 6: 27.

75. Le Port A, Cottrell G, Dechavanne C, Briand V, Bouraima A, et al. (2011) Prevention of malaria during pregnancy: assessing the effect of the distribution of IPTp through the national policy in Benin. Am J Trop Med Hyg 84: 270-275.

76. Sirima SB, Cotte AH, Konate A, Moran AC, Asamoa K, et al. (2006) Malaria prevention during pregnancy: assessing the disease burden one year after implementing a program of intermittent preventive treatment in Koupela District, Burkina Faso. Am J Trop Med Hyg 75: 205-211.

77. Tako EA, Zhou A, Lohoue J, Leke R, Taylor DW, et al. (2005) Risk factors for placental malaria and its effect on pregnancy outcome in Yaounde, Cameroon. Am J Trop Med Hyg 72: 236-242.

78. Salihu HM, Tchuinguem G, Ratard R (2000) Effect of chloroquine prophylaxis on birthweight and malaria parasite load among pregnant women delivering in a regional hospital in Cameroon. West Indian Med J 49: 143-147.

79. Akum AE, Kuoh AJ, Minang JT, Achimbom BM, Ahmadou MJ, et al. (2005) The effect of maternal, umbilical cord and placental malaria parasitaemia on the birthweight of newborns from South-western Cameroon. Acta Paediatr 94: 917-923.

80. Wessel H, Cnattingius S, Bergstrom S, Dupret A, Reitmaier P (1996) Maternal risk factors for preterm birth and low birthweight in Cape Verde. Acta Obstet Gynecol Scand 75: 360-366.

81. Vanga-Bosson HA, Coffie PA, Kanhon S, Sloan C, Kouakou F, et al. (2011) Coverage of intermittent prevention treatment with sulphadoxine-pyrimethamine among pregnant women and congenital malaria in Cote d'Ivoire. Malar J 10: 105.

82. Rayco-Solon P, Fulford AJ, Prentice AM (2005) Differential effects of seasonality on preterm birth and intrauterine growth restriction in rural Africans. Am J Clin Nutr 81: 134-139.

83. Okoko JB, Wesumperuma HL, Hart CA (2001) The influence of prematurity and low birthweight on transplacental antibody transfer in a rural West African population. Trop Med Int Health 6: 529-534.

84. Klufio CA, Lassey AT, Annan BD, Wilson JB (2001) Birthweight distribution at Korle-Bu Teaching Hospital, Ghana. East Afr Med J 78: 418-423.

85. Shuaib FM, Jolly PE, Ehiri JE, Yatich N, Jiang Y, et al. (2010) Association between birth outcomes and aflatoxin B1 biomarker blood levels in pregnant women in Kumasi, Ghana. Trop Med Int Health 15: 160-167.

86. Oduro AR, Fryauff DJ, Koram KA, Rogers WO, Anto F, et al. (2010) Sulfadoxine-pyrimethamine-based intermittent preventive treatment, bed net use, and antenatal care during pregnancy: demographic trends and impact on the health of newborns in the Kassena Nankana District, northeastern Ghana. Am J Trop Med Hyg 83: 79-89.

87. Roth A, Jensen H, Garly ML, Djana Q, Martins CL, et al. (2004) Low birth weight infants and Calmette-Guerin bacillus vaccination at birth: community study from Guinea-Bissau. Pediatr Infect Dis J 23: 544-550.

88. Sodemann M, Nielsen J, Veirum J, Jakobsen MS, Biai S, et al. (2008) Hypothermia of newborns is associated with excess mortality in the first 2 months of life in Guinea-Bissau, West Africa. Trop Med Int Health 13: 980-986.

89. Kayentao K, Kodio M, Newman RD, Maiga H, Doumtabe D, et al. (2005) Comparison of intermittent preventive treatment with chemoprophylaxis for the prevention of malaria during pregnancy in Mali. J Infect Dis 191: 109-116.

90. Famanta A, Diakite M, Diawara SI, Diakite SA, Doumbia S, et al. (2011) [Prevalence of maternal and placental malaria and of neonatal low birth weight in a semi-urban area of Bamako (Mali)]. Sante 21: 3-7.

91. Onah HE (2003) Effect of prolongation of pregnancy on perinatal mortality. Int J Gynaecol Obstet 80: 255-261.

92. Egwunyenga OA, Ajayi JA, Popova-Duhlinska DD, Nmorsi OP (1996) Malaria infection of the cord and birthweights in Nigerians. Cent Afr J Med 42: 265-268.

93. Lawoyin TO (2007) Infant and maternal deaths in rural south west Nigeria: a prospective study. Afr J Med Med Sci 36: 235-241.

94. Ezeaka VC, Egri-Okwaji MT, Renner JK, Grange AO (2003) Anthropometric measurements in the detection of low birth weight infants in Lagos. Niger Postgrad Med J 10: 168-172.

95. Ozumba BC, Okafor HU (2004) Low-birthweight babies in Eastern Nigeria. Public Health 118: 244-246.

96. Onah HE (2000) Declining fetal growth standards in Enugu, Nigeria. Int J Gynaecol Obstet 68: 219-224.

97. Onyiriuka AN (2006) Trends in incidence of delivery of low birth weight infants in Benin City, southern Nigeria. Niger Postgrad Med J 13: 189-194.

98. Ibekwe PC, Dimejesi IB (2008) Obstetric indices at the Ebonyi State University Teaching Hospital, Abakaliki, South East Nigeria. Niger J Med 17: 399-402.

99. Ekure EN, Ezeaka VC, Iroha E, Egri-Okwaji M (2011) Prospective audit of perinatal mortality among inborn babies in a tertiary health center in Lagos, Nigeria. Niger J Clin Pract 14: 88-94.

100. Falade CO, Yusuf BO, Fadero FF, Mokuolu OA, Hamer DH, et al. (2007) Intermittent preventive treatment with sulphadoxine-pyrimethamine is effective in preventing maternal and placental malaria in Ibadan, south-western Nigeria. Malar J 6: 88.

101. Fawole AO, Shah A, Tongo O, Dara K, El-Ladan AM, et al. (2011) Determinants of perinatal mortality in Nigeria. Int J Gynaecol Obstet 114: 37-42.

102. Aribodor DN, Nwaorgu OC, Eneanya CI, Okoli I, Pukkila-Worley R, et al. (2009) Association of low birth weight and placental malarial infection in Nigeria. J Infect Dev Ctries 3: 620-623.

103. Sotimehin SA, Runsewe-Abiodun TI, Oladapo OT, Njokanma OF, Olanrewaju DM (2008) Possible risk factors for congenital malaria at a tertiary care hospital in Sagamu, Ogun State, South-West Nigeria. J Trop Pediatr 54: 313-320.

104. Olowe SA (1981) Standards of intrauterine growth for an African population at sea level. J Pediatr 99: 489-495.

105. Tongo OO, Orimadegun AE, Akinyinka OO (2011) Utilisation of malaria preventive measures during pregnancy and birth outcomes in Ibadan, Nigeria. BMC Pregnancy Childbirth 11: 60.

106. Sule-Odu AO, Ogunledun A, Olatunji AO (2002) Impact of asymptomatic maternal malaria parasitaemia at parturition on perinatal outcome. J Obstet Gynaecol 22: 25-28.

107. Akanbi OM, Odaibo AB, Ademowo OG (2009) The burden of malaria infection on pregnant women and birth weight of infants in south western Nigeria. East Afr J Public Health 6: 63-68.

108. Akpala CO (1993) Perinatal mortality in a northern Nigerian rural community. J R Soc Health 113: 124-127.

109. Camara B, Diack B, Diouf S, Signate-Sy H, Sall MG, et al. (1995) [Low birth weight: rate and risk factors in the Guedianwaye district (suburb of Dakar, Senegal)]. Dakar Med 40: 213-219.

110. Olliaro PL, Delenne H, Cisse M, Badiane M, Olliaro A, et al. (2008) Implementation of intermittent preventive treatment in pregnancy with sulphadoxine/pyrimethamine (IPTp-SP) at a district health centre in rural Senegal. Malar J 7: 234.

111. Gueye M, Gueye-Diagne NR, Faye PM, Seye PI, Ndiaye O (2011) [Screening of low-birthweight babies with calf circumference measurement. Study of a newborn population in the Abass Ndao Hospital Center of Dakar (Senegal)]. Arch Pediatr 18: 1113-1114.

112. Balaka B, Baeta S, Agbere AD, Boko K, Kessie K, et al. (2002) [Risk factors associated with prematurity at the University Hospital of Lome, Togo]. Bull Soc Pathol Exot 95: 280-283.
